# Comparison between chaotropic and detergent‐based sample preparation workflow in tendon for mass spectrometry analysis

**DOI:** 10.1002/pmic.201700018

**Published:** 2017-06-22

**Authors:** Yalda Ashraf Kharaz, Danae Zamboulis, Karen Sanders, Eithne Comerford, Peter Clegg, Mandy Peffers

**Affiliations:** ^1^ Department of Musculoskeletal Biology, Institute of Ageing and Chronic Disease University of Liverpool Liverpool UK; ^2^ The MRC‐Arthritis Research UK Centre for Integrated research into Musculoskeletal Ageing (CIMA) Liverpool UK

**Keywords:** Guanidine‐Hcl, Proteomics, Rapigest™, Tendon, Urea

## Abstract

Exploring the tendon proteome is a challenging but important task for understanding the mechanisms of physiological/pathological processes during ageing and disease and for the development of new treatments. Several extraction methods have been utilised for tendon mass spectrometry, however different extraction methods have not been simultaneously compared. In the present study we compared protein extraction in tendon with two chaotropic agents, guanidine hydrochloride (GnHCl) and urea, a detergent, RapiGest™, and their combinations for shotgun mass spectrometry. An initial proteomic analysis was performed following urea, GnHCl, and RapiGest™ extraction of equine superficial digital flexor tendon (SDFT) tissue. Subsequently, another proteomic analysis was performed following extraction with GnHCl, Rapigest™, and their combinations. Between the two chaotropic agents, GnHCl extracted more proteins, whilst a greater number of proteins were solely identified after Rapigest™ extraction. Protein extraction with a combination of GnHCl followed by RapiGest™ on the insoluble pellet demonstrated, after label‐free quantification, increased abundance of identified collagen proteins and low sample to sample variability. In contrast, GnHCl extraction on its own showed increased abundance of identified proteoglycans and cellular proteins. Therefore, the selection of protein extraction method for tendon tissue for mass spectrometry analysis should reflect the focus of the study.

**Abbreviations**
Ambic: ammonium bicarbonate
ECM: extracellular matrix
HCD: high energy collisional dissociation
LF: label‐free
GnHCl: guanidine hydrochloride
PANTHER: protein analysis through evolutionary relationships
SDFT: superficial digital flexor tendon

## 1 Introduction

Tendons are dense connective tissues that perform key roles in the musculoskeletal system. They serve primarily to transfer the pull of muscles to bone 1 but also engage in locomotion by transferring the forces generated by the muscles to the skeleton 2. Injuries to tendons are common in humans as well as other species such as the horse 3, 4. More than 30 million tendon injuries per year are reported worldwide in humans 5 and represents a major health care burden 6. Among tendons, the rotator cuff, Achilles, and patellar tendons are the most commonly affected in humans 7, whilst in the horse the superficial digital flexor tendon (SDFT) is most commonly injured 4. Risk factors such as repetitive loading 8, chronic inflammation 9, genetic factors 10, and ageing 11 have been demonstrated to play a role in tendon injury in both humans and the horse.

**Significance of the study**

The ability to produce robust and effective methodologies in mass spectrometry‐based proteomic sample preparation will be invaluable for future studies in tendon enabling its comprehensive proteomic characterisation and helping to identify potential target areas for diagnostics/therapeutic purposes. In this study, we determine the first comparison between chaotropic‐ and detergent‐based work‐flows for tendon sample preparation for mass spectrometry analysis.

Mass spectrometry (MS) analysis is becoming an attractive tool for tendon proteomic profile characterisation, with studies outlining the differences in disease 9, with ageing 11, at different anatomical compartement 12, as well as differences between tendon and ligament and tissues engineered tissues 13. However, a robust and reproducible protein extraction method specific for tendon tissue is lacking. The use of proteomic techniques will allow tendon protein profiles and patterns to be defined under different conditions to obtain a clearer insight into tendon composition, which will have an impact for cell based therapies and tissue engineering strategies in tendon disease.

Tendons are composed of water, a small population of cells (predominately tenocytes) and extracellular matrix (ECM) consisting predominately of collagens (I, III, V, VI, XII, XIV) 8, 14, proteoglycans 15, glycoproteins 14, and elastin 16. Tendon protein extraction can be challenging due to its collagen‐rich composition which exhibits high intermolecular cross‐linking 17 that accumulates with ageing and therefore makes it resistant to extraction 11. Chaotropic agents 11, 13, surfactants 12, 18, 19, and other agents such as cyanogen bromide and proteases 20 have been used for tendon protein extraction for MS analysis. However, to date no study has compared different extraction methods for tendon.

Guanidine‐HCl (GnHCl) is one of the most efficient chaotropic agents 21 and has been used for protein extraction in ECM‐rich tissues such as bone 22, 23, cartilage 24, 25, ligament and tendon 13, 20, 26. Urea is another chaotropic agent that has been widely used and is efficient for cell and tissue lysis 27, 28, 29. Chaotropic agents exert their function by extracting non‐covalently bound ECM and cellular proteins leaving behind an insoluble fraction 11, 13, 25. Another extraction technique that has recently been used in tendon proteomic studies is the surfactant RapiGest™ (Waters) 12, 18, which was shown to increase protein identification 25, 30.

The aim of this study was (i) to compare differences between two different chaotropic agents and a detergent based work‐flow for label‐free (LF) MS‐based analysis in tendon and (ii) to identify whether combining a chaotropic agent and a detergent for protein extraction could increase the protein coverage in tendon.

## 2 Materials and methods

All chemicals were supplied by Sigma–Aldrich, Dorset, UK unless otherwise stated.

### 2.1 Tissue collection and preparation

Forelimbs, distal to the carpus, were collected from middle‐aged horses (*n* = 3, aged 12.3 ± 1.15, mean ± SD) from a commercial equine abattoir. Ethical approval is not required for samples collected as by‐products of the agricultural industry according to the Animal (Scientific Procedures) Act 1986, Schedule 2. SDFTs were collected and a 2 cm sample was dissected from the mid‐metacarpal region. Only tendons that had no evidence of previous tendon injury at post‐mortem examination were included in the study. All samples were snap‐frozen in liquid nitrogen and stored at −80°C until required.

Samples for protein extraction were homogenised using a dismembrator (B.Braun Biotech. International, Germany), split into three or four aliquots of approximately 20 mg and deglycolysated with 1 U/ml chondroitinase ABC for 6 h at 37°C 13 prior to protein extraction.

### 2.2 Comparison between GnHCl, urea and RapiGest™ extraction

#### 2.2.1 GnHCl protein extraction

0.5 mL of GnHCl extraction buffer (4 M GnHCl, 65 mM dithiothreitol (DTT), 50 mM sodium acetate and protease inhibitors (complete Protease Inhibitors, EDTA‐free, Roche Applied Science) was added to the samples. Each sample was then sonicated on ice (three cycles of 10 s each at 40% output) on an ultrasonic processor followed by incubation at 4°C on a shaker for 48 h. After centrifugation at 15 000 rpm at 4°C for 15 min, the supernatant (soluble fraction) was collected and stored at −20°C until further analysis.

#### 2.2.2 Urea protein extraction

0.5 mL of urea extraction buffer (7 M Urea, 0.15 M Sodium Chloride, 50 mM tris hydrochloride and protease inhibitors, pH 6.5) was added to each sample followed by sonication, centrifugation, and supernatant collection as described in Section 2.2.1.

#### 2.2.3 RapiGest™ protein extraction

The 250 μL 50 mM ammonium bicarbonate (Ambic) was added to each sample before sonication (Section 2.2.1). The samples were then topped up with 250 μL 0.2% RapiGest™ in 50 mM Ambic and heated at 80°C for 10 min. A second heating step at 60°C for 1 h was performed after the samples were left to cool down at room temperature for 10 min. Following the second heating step, the samples were spun down at maximum speed for 10 min and the supernatant was collected. The remaining pellet was topped up with 20 μL RapiGest™ (0.1%, in 50 mM Ambic) and heated at 60°C for 10 min before centrifugation at maximum speed for 10 min. The supernatant was collected and added to the previously collected supernatant (soluble fraction).

### 2.3 Comparison between GnHCl and RapiGest™ extraction and their combinations

**Figure 1:**
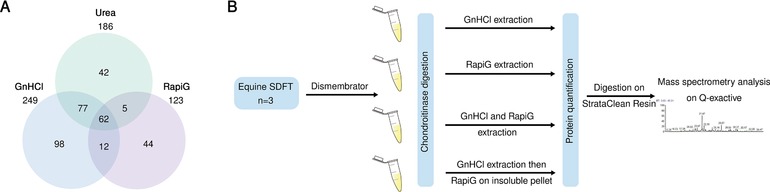
(A) Venn diagram of GnHCl, urea, and RapiGest™ extraction methods. Total number, common, and unique proteins identified following MS. All identified proteins in each method are can be found in Supporting Information Table 1. (B) Schematic workflow of follow up experiment using the chaotropic agent GnHCl, the surfactant RapiGest™, a combination of GnHCl and RapiGest™, and a combination of GnHCl followed by RapiGest™ extraction on the insoluble pellet.

A schematic representation of the experimental workflow for the comparison of GnHCl and RapiGest extraction methods and their combinations is presented in Fig. 1B.

#### 2.3.1 GnHCl and Rapigest combined protein extraction

450 μL GnHCl and RapiGest™ extraction buffer (0.1% RapiGest™ in guanidine extraction buffer) was added to each sample before sonication (Section 2.2.1). The samples were then incubated on a shaker for 48 h at 4°C followed by heating at 80°C for 10 min. A second heating step at 60°C for 1 h was performed after the samples were left to cool down at room temperature for 10 min. Following the second heating step, the samples were spun down at maximum speed for 10 min at room temperature, and the supernatant was collected. The remaining pellet was topped up with 20 μL RapiGest™ (0.1%, in 50 mM Ambic), heated at 60°C for 10 min, centrifuged, and the supernatant collected as described in Section 2.2.3.

#### 2.3.2 GnHCl extraction followed by RapiGest™ extraction on the insoluble pellet

The 250 μL GnHCl extraction buffer was added to each sample before sonication (Section 2.2). The samples were then incubated on a shaker for 48 h at 4°C, centrifuged and the soluble fraction collected (Section 2.2.1). Subsequently, the remaining pellet was washed three times with 100 μL 50 mM Ambic. The supernatant from the first wash was collected and added to the soluble fraction (additional washes were discarded). 250 μL 0.2% RapiGest™ was added to the insoluble pellet and the RapiGest™ extraction steps undertaken as described in Section 2.2.3.

The same protocol was used when comparing different concentrations of RapiGest™ (0.1, 0.2 and 0.4%) following GnHCl extraction.

### 2.4 In‐solution trypsin digestion and LC‐MS/MS

Prior to trypsin digestion the protein concentration of each soluble fraction was calculated using the Pierce™ 660 nm protein assay and samples were analysed by 1D‐SDS‐PAGE gel electrophoresis to assess gross qualitative differences in protein profiles. In‐solution tryptic digest was carried out on 10 μL of strataclean™ resins (Agilent Genomics, UK) on 100 μg protein for each sample. Prior to digest Strataclean beads were washed 3× of 25 mM ambic. In‐solution tryptic digestion of protein samples was carried out following sequential reduction and alkylation in 3 mM DTT (60°C for 10 min) and then 9 mM iodoacetamide (30 min in the dark at room temperature) with trypsin at a ratio of 1:25 protein: trypsin ratio overnight at 37°C. Detergent inactivation was then assumed by incubating for 45 min at 37°C with trifluoroacetic acid (VWR International, UK) to a final concentration of 0.5% *(v/v)*. Following centrifugation (10 min, 15 000 × *g*) the soluble phase was retrieved 31. LC‐MS/MS was performed using an Ultimate 3000 nano system (Dionex/Thermo Fischer) coupled online to a Q‐Exactive Quadrupole‐Orbitrap mass spectrometer. 50 ng tryptic peptides from randomised samples was loaded onto the column on a one h gradient with an inter‐sample 30 min blank 26.

### 2.5 Proteomic data analysis

MS data were analysed for protein identification using PEAKS (version, 7, Bioinformatics Solution, Waterloo, Canada) and label‐free (LF) quantification was performed with Progenesis^QI^ LC‐MS (Waters, Elstree Hertfordshire, UK) software 26. The MS data has been deposited in PRIDE database (http://www.ebi.ac.uk/pride/) at the European Bioinformatics Institute under the accession number PXD004453.

#### 2.5.1 Protein identification

Raw MS/MS files were imported into PEAKS studio 7 (Bioinformatics solution, 7, Waterloo, Canada) and searched against the UniHorse database (http://www.uniprot.org/proteomes/). Search parameters used were: 10 ppm peptide mass tolerance and 0.01 Da fragment mass tolerance; precursor mass search type, monoisotopic; enzyme, trypsin; max missed cleavage, 1; nonspecific cleavage, 1; fixed modification; carbamidomethylation; variable modifications, methionine oxidation and hydroxylation; variable PTMs per peptide, 3. Search results were adjusted to 1% FDR at peptide spectrum matches, –10lg*p > *20, unique peptides ≥2, and confidence score ≥50%.

#### 2.5.2 GO and protein network analysis

Identified proteins for each extraction method and were classified into ECM categories according to Matrisome Project 32 and for cellular compartments according through PANTHER (protein analysis through evolutionary relationships) 33.

#### 2.5.3 LF quantification

LF quantitative analysis between different extraction methods was performed using Progenesis^QI^ LC‐MS as previously described 26. Briefly, the top five spectra for each feature were exported from Progenesis^QI^ and utilized for peptide identification with a PEAKS studio 7 searching against the UniHorse database. Search parameters used were as decribed in Section 2.5.1 and were re‐imported into Progenesis^QI^. Differentially abundant proteins (*p* <0.05, fold change >2) in each group were categorised through PANTHER Classification System.

#### 2.5.4 Statistical analysis

Statistical analysis was performed on protein concentration measurements using one‐way analysis of variance (ANOVA) with Bonferroni post‐hoc test using Graphpad Prism (version 6, Graphpad Sofware, La Jolla California, USA). Statistical analysis for LF quantification was performed by Progenesis^QI^ software on all detected features using transformed normalized abundance for ANOVA. Identified proteins with two or more peptides, greater than 2 fold abundance and with a *p*‐value adjusted to FDR *p* < 0.05 were considered as significantly differentially abundant.

## 3 Results

### 3.1 A higher number of proteins were identified with GnHCl in comparison to Urea and RapiGest™ extraction methods

A total of 249, 186, and 123 proteins were identified with GnHCl, Urea, and RapiGest™ extraction methods respectively. Between all three extraction methods 62 proteins were found to be common (Fig. 1A). Chaotropic agents identified 139 proteins in common, but a higher number of total and unique proteins were indentified in GnHCl compared to Urea (Fig. 1A). RapiGest™ extraction gave less common identified proteins with GnHCl (74) and with urea (67), whilst having more unique proteins than urea (Fig. 1A). Based on these results, a combination of GnHCl and RapiGest™ was investigated (Fig. 1B). All identified proteins in the three methods are provided in Supporting Information Table 1.

### 3.2 Improved extraction efficiency and less disparity was found with the extraction method of GnHCl followed by RapiGest™ on the insoluble pellet

**Figure 2:**
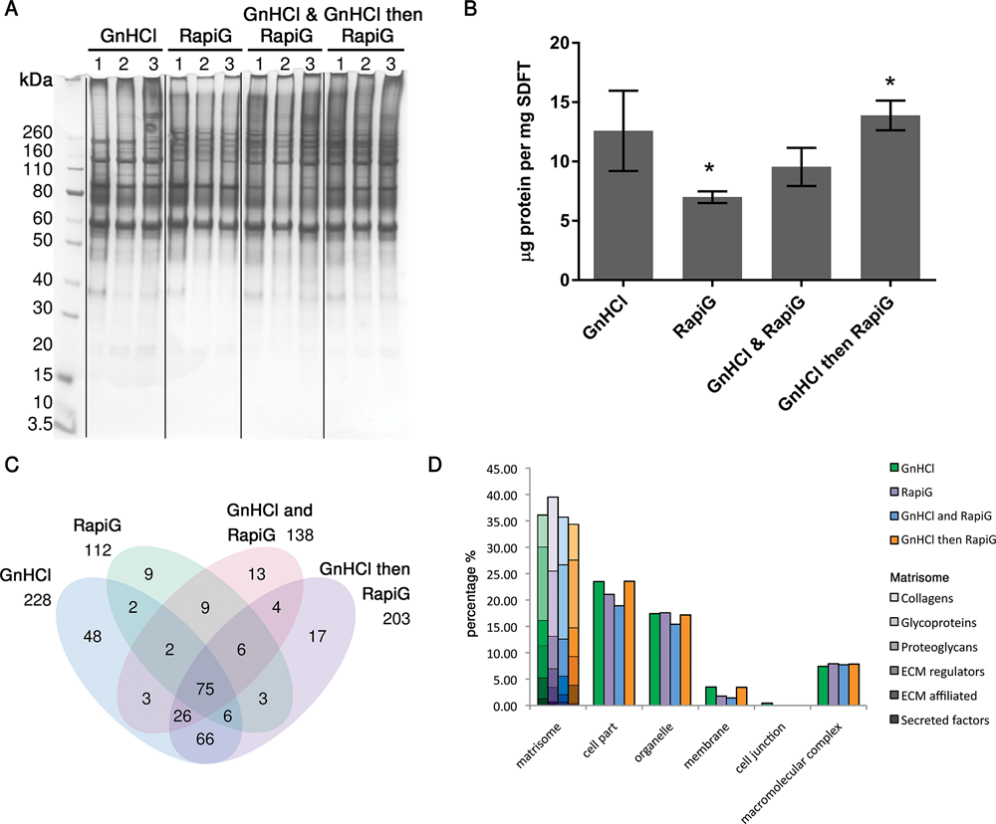
(A) 1D SDS‐PAGE analysis of the protein profiles of GnHCl, RapiGest™, GnHCl and RapiGest™, and GnHCl followed by RapiGest™ extraction methods. (B) Protein concentration yielded with the different extraction methods. Values are mean and error bars represent SD, *p < 0.05. (C) Venn diagram of the different extraction methods. (D) Classification of identified proteins for each extraction according to cell compartment (PANTHER) and matrisome classifcation (Matrisome Project). All identified proteins in each method can be found in Supporting Information Table 2.

1D SDS‐PAGE analysis of GnHCl and RapiGest™ extraction and their combinations displayed variability in protein profiles with the RapiGest™ extraction showing an absence of high molecular proteins compared to the other methods (Fig. 2A). GnHCl followed by RapiGest™ extraction yielded a higher protein concentration (13.9 ± 1.2 μg/mg weight) with the least variability between the samples in comparison to the other three extraction methods (Fig. 2B). A total number of 229, 112, 138, and 203 proteins were identified for GnHCl, RapiGest™, GnHCl and RapiGest™ and GnHCl followed by RapiGest™ respectively (Fig. 2C). The GnHCl followed by RapiGest method gave the most identified unique proteins whilst RapiGest™ had the least (Fig. 2C). Protein composition with GnHCl and GnHCl followed by RapiGest™ extracts revealed a similar percentage of cell associated proteins (49%) which was higher than for the other two methods. Following RapiGest™ extraction, a higher percentage of proteins (39%) associated to the matrisome was identified being predominantly core matrisomal collagens (Fig. 2D). All identified proteins in the four methods are provided in Supporting Information Table 2.

**Figure 3:**
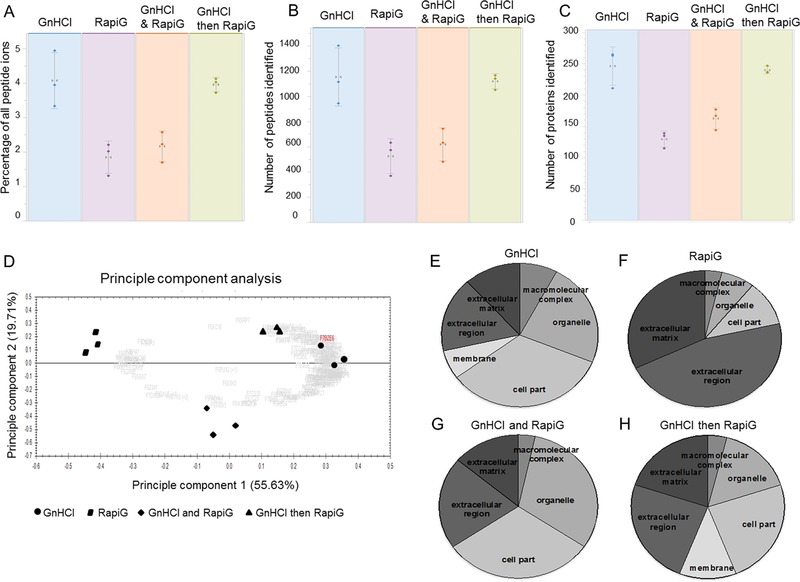
(A‐C) Quality control and label free quantitative analysis comparison between GnHCl, RapiGest™, GnHCl and Rapigest™, and GnHCl followed by RapiGest™ using Progenesis^QI^ software. The variation in percentage of all peptide ions (A), number of peptides (B) and proteins (C) was presented for each extraction method. (D) PCA plot of all methods, GnHCl followed by RapiGest™ samples grouped closer together. (E‐H) Significantly abundant proteins (fold change >2 and *p <* 0.05) identified in GnHCl (E), RapiGest (F), GnHCl and RapiGest™ (G), and GnHCl followed by RapiGest™ (H) extraction. Abundant proteins in each group were categorised using PANTHER classification.

The Progenesis^QI^ quality control report demonstrated less inter‐sample variability in percentage of peptide ions and number of peptides and proteins for the GnHCl followed by RapiGest™ extraction method (Fig. 3A–C). This finding was supported by the protein PCA plot which demonstrated that samples from the GnHCl followed by RapiGest™ extraction method were grouped closer together (Fig. 3D). LF analysis demonstrated a set of 170 proteins within the four extraction methods with a fold change >2, unique peptides *≥*2, and FDR adjusted *p* < 0.05. From the proteins that were most abundant in the GnHCl extract, 65% were cellular and intracellular associated, with the remaining 28 and 6% identified as ECM and membrane bound proteins respectively (Fig. 3E). In contrast, from the proteins that were most abundant after RapiGest™ extraction, a considerably higher percentage were ECM associated proteins (78%) and a smaller percentage were cell associated proteins (22%) (Fig. 3F). Of the most abundant proteins in GnHCl and RapiGest™ extraction, 65% and 35% were ECM and cellular associated proteins respectively (Fig. 3G). The most abundant proteins in the GnHCl followed by RapiGest™ extract were classified as 44% ECM associated, 44% cellular associated and the remaining 12% were membrane associated proteins (Fig. 3H).

### 3.3 Quantitative differences in protein composition were observed between the GnHCl and GnHCl followed by RapiGest extraction methods

**Figure 4:**
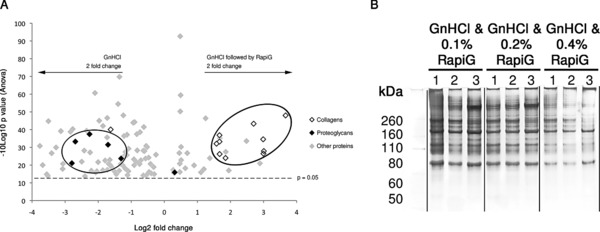
(A) The volcano plot demonstrates all differentially abundant proteins between GnHCl and GnHCl followed by RapiGest™ (fold change >2 and *p <* 0.05). Collagens were most abundantly identified in GnHCl followed by RapiGest™ and proteoglycans in GnHCl extraction. All differentially abundant proteins between GnHCl and GnHCl followed by RapiGest™ can be found in Supporting Information Table 3. (B) 1D SDS‐PAGE analysis of GnHCl followed by different concentrations of RapiGest™.

Subsequent relative protein abundance between GnHCl and GnHCl followed by RapiGest™ was assessed by LF quantitative analysis. There were 35 differentially abundant proteins identified with two or more peptides, a *p*‐value < 0.05, and more than a 2‐fold change (Fig. 4A). Collagens, such as collagen type I alpha 2 chain, collagen type II alpha 1 chain and collagen type V alpha 1, were most abundantly found in the GnHCl followed by RapiGest™ extraction (Table 1). On the other hand, proteoglycans, such as fibromodulin and lumican, were most abundantly found after GnHCl extraction (Table 1). Several cellular proteins, such as actin 1 and 4, talin 1, and tubulin alpha 4A, were also found to be more abundant after GnHCl extraction. Following GnHCl extraction, the collagenous proteins and the proteoglycans abundance represented 49% and 20% respectively of the overall identified proteins abundance. Whereas GnHCl followed by RapiGest™ extraction resulted in 80 and 4% abundance of collagens and proteoglycans respectively (Supporting Information Table 3).

**Table 1:** A select number of significantly differentially abundant ECM proteins identified between GnHCl and GnHCl followed by RapiGest extraction by Progenesis^QI^ LC‐MS software (>2‐fold change, *p* < 0.05, ≥2 peptides)

Higest mean condition	Accession	Description	Peptide count	Max fold change	ANOVA (*p*)
**GnHCl**	F6PVJ6	Osteoglycin	8	5.8	0.008
	A2Q126	Fibromodulin	6	4.03	0.0004
	F6SKT2	Lumincan	11	5.4	0.0002
	O46542	Decorin	19	2.73	0.0007
	O46403	Biglycan	16	2.08	0.004
	F6YR34	Thrombospondin 1	14	4.50	0.0015
**GnHCl followed by RapiGest**	F6RTH9	Collagen type I alpha 2	126	10.67	1.58E‐05
	F6R4Y3	Collagen type I alpha 1	134	2.51	0.004
	F6XIM5	Collagen type II alpha 1	31	2.67	0.0002
	F6R4Y3	Collagen type III alpha 1	105	3.03	0.004
	F6VVM5	Collagen type IV alpha 1	5	5.46	4.64E‐05
	F6Q0M8	Collagen type IV alpha 2	6	2.68	0.002
	F6PLH0	Collagen type V alpha 1	21	6.77	0.002
	F7BH47	Collagen type V alpha 2	30	6.67	0.0004
	F6XKF5	Collagen type V alpha 3	6	2.673	0.0005
	F6XHX	Collagen type XVIII alpha 1	3	6.71	0.002

The addition of RapiGest™ at variable concentrations (0.1, 0.2 and 0.4%) following GnHCl extraction demonstrated no differences on 1D SDS‐PAGE analysis between the three concentrations indicating the use of 0.1% RapiGest™ on the insoluble pellet is sufficient (Fig. 4B).

## 4 Discussion

This is the first study to compare different protein extraction methods in tendon for shotgun MS. The experimental design involved a proteomics comparison between GnHCl, urea, and Rapigest™ extractions and a further comparison between GnHCl and Rapigest™ and their combinations.

Both GnHCl and urea are chaotropic agents implying that they disrupt the non‐covalent bonds within the proteins tertiary structure. In this study, we found GnHCl extraction increased the number of proteins identified compared to urea. The higher number of intracellular proteins extracted with GnHCl indicates that this agent is able to disrupt cellular membranes in a more efficient manner, which is consistent with other studies findings, whereas GnHCl was thought to disrupt the cell membrane causing permeabilisation 34, 35. In tendon, the relatively poorer protein identification by urea could possibly be due to its lower ability to solubilise the lipid bilayer of membranes which could lead to reduced release of cell contents and fewer less abundant proteins.

The surfactant Rapigest™ was chosen for the detergent‐based extraction method, as it does not supress peptide ionisation or modify peptides and proteins making it compatible with MS 36 also offering a simple extraction method for tendon tissue. In this study, when compared to GnHCl and urea, RapiGest™ gave less overall protein identifications but had fewer proteins in common with the chaotropic agents suggesting extraction of different proteins. RapiGest™ has been shown to improve ECM MS protein coverage by its addition to the trypsin digestion solution and protein extraction efficiency in cartilage 25, 37. Based on the above findings, combinations of GnHCl and RapiGest™ extraction were further investigated in the current study.

Overall, GnHCl and GnHCl followed by RapiGest™ demonstrated a more robust extraction of tendon by yielding a higher protein concentration, more protein identifications and a good representation of cell and membrane associated proteins. In addition, GnHCl followed by RapiGest™ demonstrated the least inter‐sample variation in terms of protein concentration and in peptide and protein identification and quantification, suggesting this method to be the most consistent.

Furthermore, the addition of RapiGest™ to GnHCl extraction and RapiGest™ alone resulted in an increased abundance of identified collagens compared to GnHCl alone. Following GnHCl extraction, the proteoglycans abundance represented 20% of the overall identified protein abundance, whilst with GnHCl followed by RapiGest™ extraction it was only 4 and 80% were collagens compared to only 49% collagen proteins in GnHCl extraction. Since tendon ECM composition consists of 60–85% collagens and 1–5% proteoglycans (per dry weight tissue), GnHCl followed by RapiGest™ extraction most closely reflected the expected tendon ECM composition 38. These results thus demonstrate the advantages of combining the surfactant RapiGest™ to GnHCl extraction. However, for studies specifically looking at proteoglycans or less abundant proteins in tendon, GnHCl extraction may be more advantageous. Also for studies that are trying achieve to complete tendon proteome indentification, separate MS analysis of successive fractions of GnHCl followed by Rapigest™ method could provide more identifications. In addition, the tendon has different anatomical compartments with distinct proteome composition 12 and studies looking at a specific compartment such as the interfascicular matrix 12 might benefit from one method over the other.

In our study, elastin was not identified following any of the extraction methods used. This may be due to elastin being highly cross‐linked, hydrophobic 39 and containing repetitive sequences 40. In order to identify elastin, other mass spectrometry studies have used cyanogen bromide extraction 41 and elastase digestion 20, 40 or 2D separation techniques which could be applied in future studies.

In conclusion, we have shown for the first time a comparison between different extraction methods for MS analysis in tendon tissue. Whilst GnHCl followed by RapiGest™ extraction may be appropriate for extraction and identification of collagenous proteins, GnHCl extraction may be more appropriate for extraction and indentification of cellular proteins and proteoglycans in tendon tissue. Therefore, the optimal extraction method should be based on the hypothesis and study design. Our findings make a significant contribution in the field of tendon proteomics and will be invaluable for future studies in tendon research, and could benefit in the diagnosis and therapeutics of tendon disease.

*The authors have declared no conflict of interest*.

## Supporting information

Supporting Information material

Supporting Information material

Supporting Information material
